# Larger ^18^F-fluoroboronotyrosine (FBY) active volume beyond MRI contrast enhancement in diffuse gliomas than in circumscribed brain tumors

**DOI:** 10.1186/s13550-022-00896-w

**Published:** 2022-04-18

**Authors:** Ziren Kong, Zhu Li, Junyi Chen, Wenbin Ma, Yu Wang, Zhi Yang, Zhibo Liu

**Affiliations:** 1 Department of Neurosurgery, Peking Union Medical College Hospital, Chinese Academy of Medical Sciences and Peking Union Medical College, Beijing, China; 2 Department of Head and Neck Surgery, National Cancer Center/National Clinical Research Center for Cancer/Cancer Hospital, Chinese Academy of Medical Sciences and Peking Union Medical College, Beijing, China; 3 Key Laboratory of Carcinogenesis and Translational Research, Department of Nuclear Medicine, Peking University Cancer Hospital and Institute, Beijing, China; 4 Beijing National Laboratory for Molecular Sciences, Radiochemistry and Radiation Chemistry Key Laboratory of Fundamental Science, NMPA Key Laboratory for Research and Evaluation of Radiopharmaceuticals, Key Laboratory of Bioorganic Chemistry and Molecular Engineering of Ministry of Education, College of Chemistry and Molecular Engineering, Peking University, Beijing, China; 5 Peking University-Tsinghua University Center for Life Sciences, Beijing, China

**Keywords:** Fluoroboronotyrosine, PET, Brain tumor, Diffuse glioma, Metastasis, Volume comparison, Boron neutron capture therapy

## Abstract

**Background:**

To investigate the relationship between ^18^F-fluoroboronotyrosine (FBY) positron emission tomography (PET)- and magnetic resonance imaging (MRI)-defined tumor volumes in contrast-enhanced diffuse gliomas and circumscribed brain tumors.

**Methods:**

A total of 16 diffuse gliomas and 7 circumscribed brain tumors were included, and two types of three-dimensional regions of interest (ROIs), namely, MRI-based ROI (ROI_MRI_) and FBY-based ROI (ROI_FBY_), were semiautomatically defined. The overlap volume and DICE score were calculated to reveal the spatial relationship between the ROI_MRI_ and ROI_FBY_.

**Results:**

The ROI_MRI_ was smaller than the ROI_FBY_ and was mostly contained by the ROI_FBY_ with an overlap volume of 0.995 ± 0.006 in the whole population. A significant difference in the DICE score was observed between circumscribed tumors and diffuse tumors (0.886 ± 0.026 vs. 0.684 ± 0.165, *p* = 0.004), and for the regions that have increased FBY metabolism but not MRI contrast enhancement, diffuse tumors and circumscribed tumors showed similar SUVmean values (0.630 ± 0.19 vs. 0.671 ± 0.18, *p* = 0.625).

**Conclusion:**

FBY uptake beyond contrast enhancement is more significant in diffuse tumors than in circumscribed tumors, which may aid the delineation of active tumor areas and facilitate boron neutron capture therapy.

## Introduction

Diffuse gliomas, also known as diffuse astrocytic and oligodendroglial tumors, remain the most challenging oncological subjects for treatment due to tumor infiltration into the brain parenchyma, and the biological tumor boundary cannot be accurately defined with magnetic resonance imaging (MRI), which sometimes results in treatment failure [1–3]. High-grade diffuse gliomas usually display contrast enhancement on MRI [4], and the identification of tumor infiltration beyond contrast enhancement is crucial for surgical planning and adjuvant treatment [5].

^18^F-fluoroboronotyrosine (FBY) is a large neutral amino acid transporter 1 (LAT-1) targeting boron-derived tyrosine that can be easily radiolabeled through a one-step ^19^F-^18^F exchange reaction with good radiochemical yield (38 ± 14%), purity (over 99%) and metabolic stability (no defluorination or deboronation over 4 h in vitro, minimum bone uptake in vivo) [6, 7]. Previous FBY positron emission tomography (PET) studies demonstrated significant tracer uptake in high-grade gliomas with a standard uptake value (SUV) maximum (SUVmax) of 2.84 ± 0.46 and a tumor-to-normal ratio (T/N ratio) of 24.6 ± 6.3, which contributes to malignancy stratification and may facilitate boron neutron capture therapy (BNCT) due to the capability of specific tumor accumulation and minimum normal tissue exposure [7, 8]. In addition, FBY can be used for diagnosis and treatment with an identical chemical structure (the only difference is ^18^F for PET and ^19^F for BNCT) and overcome the limitation of the previous BNCT strategy in which distinct molecules were utilized for imaging and treatment [9]. However, whether FBY can be absorbed by the infiltrating area beyond contrast enhancement still needs to be determined.

This study investigates the relationship between MRI and FBY-defined tumor volume in diffuse gliomas and circumscribed brain tumors, aiming to uncover the uptake pattern of FBY and provide an alternative approach for surgical and treatment planning.

## Material and methods

### Study population

This study originated from the contrast-enhanced subpopulation of a prospective single-center study (NCT03980431) that investigated the metabolic characteristics of FBY in malignant brain tumors. Patients were recruited for FBY PET/CT if they 1) were older than 18 years of age and had a Karnofsky Performance Score greater than 70; 2) were suspected of having primary, recurrent or metastatic malignant brain tumors with planned surgery or close imaging follow-up; and 3) had no contradictions for PET/CT and MRI examinations. Patients were further included in the current analysis if significant contrast enhancement on MRI could be notified. FBY PET/CT and MRI scans were scheduled within 1 week on separate days, and pathological diagnosis was conducted in accordance with the 2016 WHO guidelines [2]. Finally, 23 patients with significant contrast-enhanced brain tumors were enrolled for this investigation, including 3 with primary WHO grade IV glioblastomas, 13 with recurrent diffuse gliomas, 5 with metastatic brain tumors, 1 with atypical meningioma and 1 with anaplastic pleomorphic xanthoastrocytoma.

### FBY PET/CT and MRI acquisition

FBY was synthesized as previously described and radiolabeled through a one-step isotope exchange reaction with a molar activity of 2.9 ± 1.4 (1.0–5.4) GBq/μmol [6]. PET/CT images were acquired using a Biograph mCT Flow 64 scanner (Siemens, Germany) 30 min after intravenous administration of FBY (3.7 MBq [0.1 mCi]/kg), with a matrix of 200 × 200 and a slice thickness of 3 mm. Images were further interpolated at the *x*-, *y*- and *z* axes as a standard protocol, which halved the physical size of each pixel at all dimensions. Standardized uptake value (SUV) map that normalized by body weight and decay factor was subsequently generated.

Thin slice contrast-enhanced T1-weighted MRI (matrix 256 × 256, slice thickness 1 mm) and T2-weighted MRI (matrix 256 × 256, slice thickness 5–6 mm) were performed with a 3.0-T Discovery MR750 scanner (GE, USA), and all images were interpolated at the x and y axes as a standard protocol. FBY PET and T2-weighted MRI were coregistered to the contrast-enhanced T1-weighted MRI data to minimize head movement between different scans.

### Tumor segmentation

Three types of regions of interest (ROIs), namely, the reference ROI (ROI_REF_), MRI-based lesion ROI (ROI_MRI_) and FBY-based lesion ROI (ROI_FBY_), were sequentially defined using 3D Slicer 4.10.2 (www.slicer.org). The ROI_REF_ was defined by placing three spherical regions on the contralateral normal cortex (mirroring the position of the tumor) to calculate the maximum SUV (Nmax) and mean SUV (Nmean) of the normal brain. The ROI_MRI_ was semiautomatically defined consistent with the current definition of gross total resection (GTR), which covers the contrast-enhanced areas for significant contrast-enhanced tumors [10].

The ROI_FBY_ was semiautomatically determined for areas with SUV/Nmax > 3.0, which is in accordance with the requirement for BNCT [11, 12]. Manual editing of the ROI_FBY_ was conducted to ensure its continuity, removing the discontinuous area from the tumor core and filling the area inside the tumor core. Structures with physiological FBY uptake (e.g., venous sinus, choroid plexus) were also manually removed.

### Volume comparison

The volumes of the ROI_MRI_ and ROI_FBY_ were calculated. The overlap volume and DICE score were calculated to reveal the spatial relationship between the ROI_MRI_ and ROI_FBY_, with the former reflecting the overlap between the two ROIs and the latter exhibiting the similarity between the two volumes:

$$\begin{aligned} & {\text{Overlap}}\;{\text{Volume}} = { }\frac{{V_{MRI} \cap V_{FBY} }}{{{\text{min}}\left( {V_{MRI} , V_{FBY} } \right)}} \\ & {\text{DICE}}\;{\text{Score}} = 2 \times { }\frac{{V_{MRI} \cap V_{FBY} }}{{V_{MRI} + V_{FBY} }} \\ \end{aligned}$$

Consistent with the 2016 WHO guidelines [1, 2], the contrast-enhanced tumors were grouped as diffuse tumors (including primary and recurrent diffuse gliomas since they grow infiltratively) and circumscribed tumors (metastatic brain tumors, meningioma and pleomorphic xanthoastrocytoma because they have a clear tumor boundary), and the differences in overlap volumes and DICE scores between the two groups were assessed by the Wilcoxon rank-sum test.

## Results

### Baseline characteristics

The characteristics of the 23 enrolled patients are detailed in Table 1. Sixteen lesions were considered to be diffuse tumors (including 3 primary WHO grade IV glioma, 1 recurrent WHO grade II glioma, 6 recurrent WHO grade III glioma, 6 recurrent WHO grade IV glioma), and 7 lesions were recognized as circumscribed tumors (including 2 metastatic tumors with breast origin, 2 metastatic tumors with lung origin, 1 metastatic tumor with colorectal origin, 1 atypical meningioma and 1 anaplastic pleomorphic xanthoastrocytoma). The Nmax and Nmean values of the normal brain were 0.116 ± 0.033 and 0.037 ± 0.016, respectively. Patients with circumscribed tumors tended to have a greater age, while the differences in sex, SUVmax, T/N ratio, SUVmean (under both the ROI_MRI_ and ROI_FBY_), tumor volume (under both the ROI_MRI_ and ROI_FBY_), Nmax and Nmean were not significant.

**Table 1:** Baseline and metabolic characteristics of the included patients

	Diffuse tumor (*n* = 16)	Circumscribed tumor (*n* = 7)	*p*
Age	39.8 ± 9.0	57.6 ± 16.0	0.025
Sex			1.000
Male	10 (62.5%)	4 (57.1%)	
Female	6 (37.5%)	3 (42.9%)	
SUVmax	2.49 ± 0.56	2.64 ± 0.31	0.507
T/N ratio	24.1 ± 7.7	21.5 ± 4.0	0.424
Nmax	0.112 ± 0.039	0.125 ± 0.017	0.395
Nmean	0.036 ± 0.018	0.039 ± 0.013	0.652
MRI parameters			
SUVmean	1.24 ± 0.25	1.21 ± 0.25	0.768
Volume	17.6 ± 21.0	25.9 ± 18.1	0.375
FBY parameters			
SUVmean	0.97 ± 0.21	1.10 ± 0.23	0.172
Volume	29.7 ± 32.1	31.5 ± 21.7	0.893

Independent sample t test or Fisher’s exact test, as appropriate, was utilized to calculate statistical significance. *SUV* standard uptake value; *T/N ratio* tumor-to-normal ratio; *Nmax* maximum SUV of normal brain; *Nmean* mean SUV of normal brain

### Volume comparison between ROI_MRI_ and ROI_FBY_

**Fig. 1:**
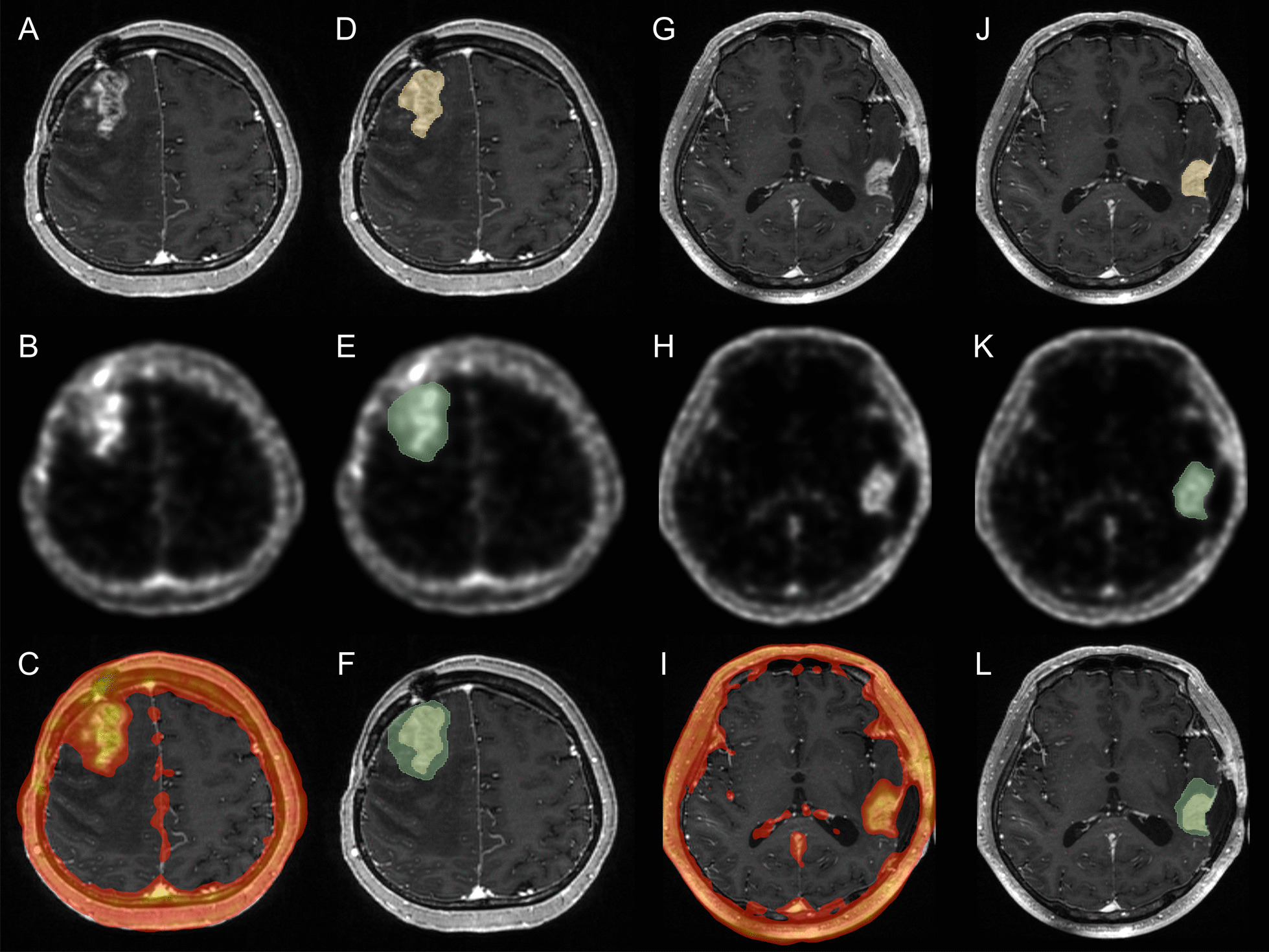
Examples of MRI and FBY PET images in diffuse tumors. A right frontal recurrent WHO grade III oligodendroglioma (IDH mutant, chromosomal 1p/19q codeletion) displayed significant contrast enhancement (**A**) and FBY activity (**B**), with increased FBY uptake seen beyond contrast enhancement in the lateral and posterior regions (**C**). Semiautomated segmentation based on MRI (**D**) and FBY PET (**E**) was performed, and nonconcentric expansion of the FBY-based segment compared with the MRI-based segment was noted (**F**). Similarly, a left temporal recurrent WHO grade IV glioblastoma (IDH-wild type) exhibited contrast enhancement (**G**) and FBY radioactivity (**H**), with the increased FBY uptake extended anteriorly and medially (**I**). The semiautomated segmentations (**J**, **K**) also verified the extension of the ROI_FBY_ to the potential invaded regions (with an abnormal T2-weighted signal). This characteristic may result from the infiltrative nature of diffuse gliomas and indicates that FBY can be absorbed in both contrast-enhanced regions and infiltrative noncontrast-enhanced tumors

**Fig. 2:**
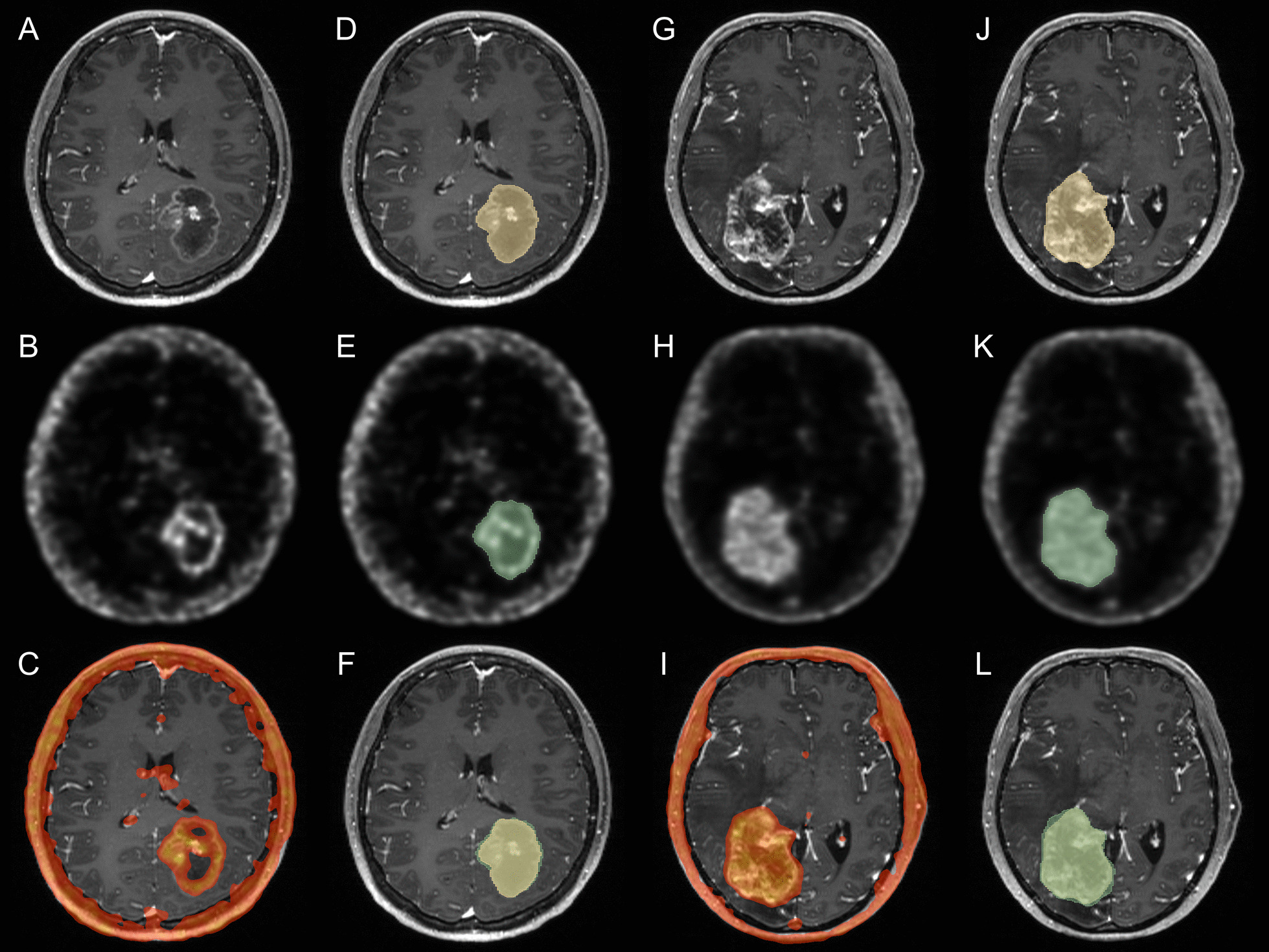
Examples of MRI and FBY PET images in circumscribed tumors. A left temporal-occipital metastatic breast cancer (HER2 positive, ER and PR negative) revealed significant contrast enhancement (**A**) and FBY activity (**B**), with better adhesion between the two regions (**C**). Semiautomated segmentation based on MRI (**D**) and FBY PET (**E**) was performed, and minimum FBY activity beyond MRI contrast enhancement was noted (**F**). Similarly, a right temporal-occipital metastatic breast cancer (HER2 negative, ER and PR positive) displayed contrast enhancement (**G**) and FBY uptake of the whole tumor region (**H**), both of which presented an explicit tumor extent (**I**). The semiautomated segmentations (**J**, **K**) were also largely equivalent (**L**), which may be due to the clear boundary of circumscribed tumors

**Fig. 3:**
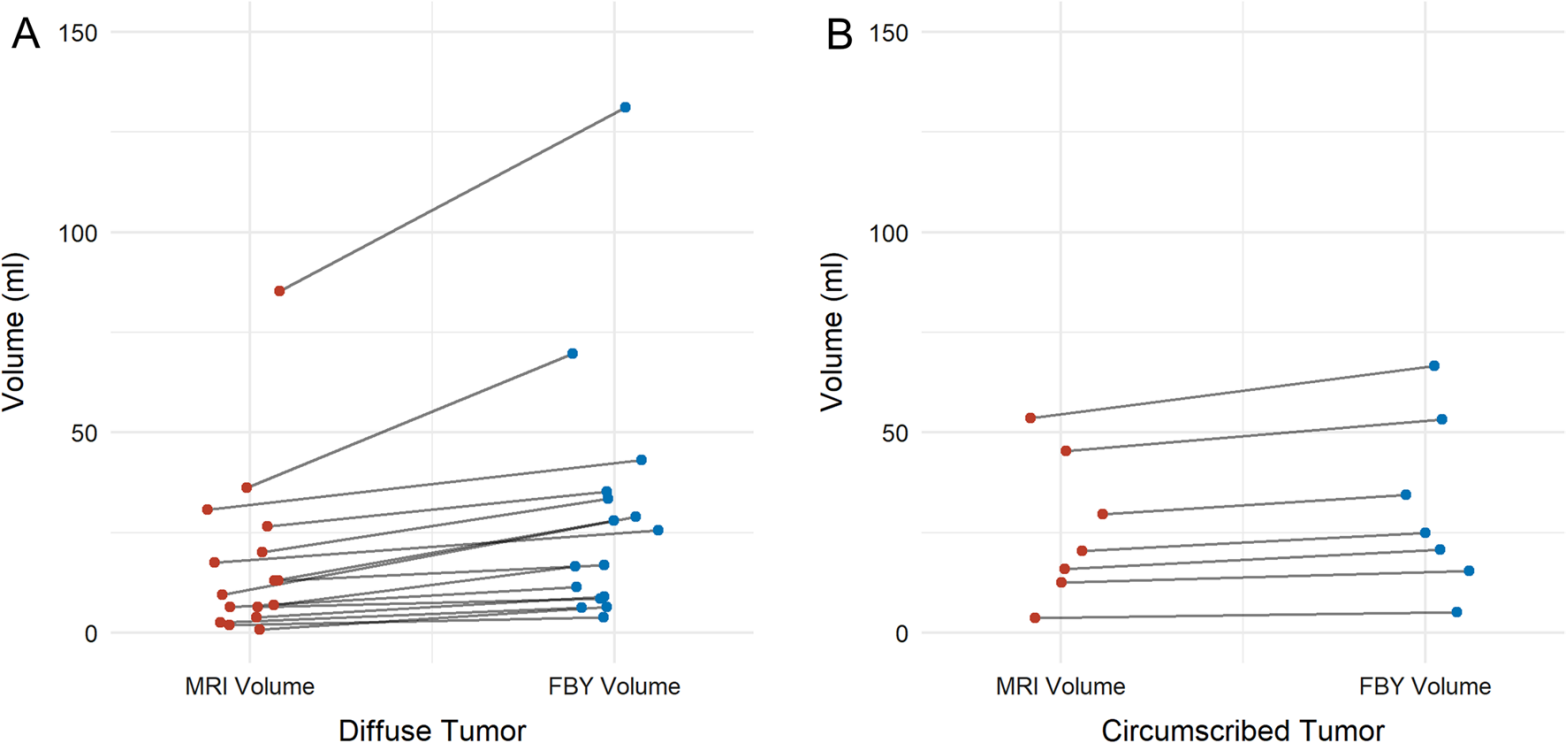
Volume comparison between diffuse tumors and circumscribed tumors. Each dot represents the individual tumor volume, with lines connecting the value of the same tumor in different segments. Red dots indicate the MRI-based volume, while blue dots represent the FBY-based volume. The diffuse tumors displayed larger volume changes between MRI-based volume and FBY-based volume than circumscribed tumors

The ROI_MRI_ was smaller than the ROI_FBY_ in all cases and was mostly contained by the ROI_FBY_ with an overlap volume of 0.995 ± 0.006 and DICE score of 0.745 ± 0.167. A significant difference in DICE scores was observed between circumscribed tumors and diffuse tumors (0.886 ± 0.026 vs. 0.684 ± 0.165, *p* = 0.004), suggesting that diffuse tumors have a larger active volume beyond contrast enhancement than circumscribed tumors; meanwhile, the differences in overlap volumes were not significant (0.996 ± 0.007 vs. 0.993 ± 0.006, *p* = 0.383). Values and examples of the ROI_MRI_- and ROI_FBY_-defined volumes are shown in Table 2, Figs. 1 and 2. Comparisons of volumes are shown in Fig. 3.

**Table 2:** Volume of MRI-based segmentation and FBY-based segmentation in contrast-enhanced brain tumor

Diffuse tumor (*n* = 16)				Circumscribed tumor (*n* = 7)			
MRI volume	FBY volume	Overlap volume	dice score	MRI volume	FBY volume	Overlap volume	DICE score
17.6 ± 21.0 ml	29.7 ± 32.1 ml	0.996 ± 0.007	0.684 ± 0.165	25.9 ± 18.1 ml	31.5 ± 21.7 ml	0.993 ± 0.006	0.886 ± 0.026

### Comparison of other metabolic parameters

In accordance with their mathematical definition, SUVmax and the T/N ratio reflect the maximum value of tumor metabolism and the ratio of SUVmax to Nmax and remained unchanged under the ROI_MRI_- and ROI_FBY_-defined volumes. The ROI_FBY_-defined SUVmean was smaller than the ROI_MRI_-defined SUVmean, and the value changes in diffuse tumors (1.24 ± 0.25 vs. 0.97 ± 0.21, *p* < 0.001 with the Wilcoxon signed-rank test) were larger than those in circumscribed tumors (1.21 ± 0.25 vs. 1.10 ± 0.23, *p* = 0.002 with the Wilcoxon signed-rank test). In the regions that have increased FBY metabolism but not MRI contrast enhancement, diffuse tumors and circumscribed tumors showed similar SUVmean values (0.630 ± 0.19 vs. 0.671 ± 0.18, *p* = 0.625 with the Wilcoxon sum-rank test) as well as the ratio of SUVmean to Nmax (5.87 ± 1.67 vs. 5.39 ± 1.43, *p* = 0.512 with the Wilcoxon sum-rank test).

## Discussion

In this study, MRI- and FBY-defined tumor volumes of 16 diffuse gliomas and 7 circumscribed brain tumors were compared. Larger metabolic volumes of FBY beyond contrast enhancement were identified in diffuse tumors, with the SUVmean in regions with increased FBY metabolism but no contrast enhancement similar to that of circumscribed tumors. This result indicated that FBY may delineate the biologically active tumor region of brain tumors, which can support future surgical planning and FBY-guided BNCT.

Compared to amino acid PET tracers (e.g., O-(2-^18^F-fluoroethyl)-L-tyrosine [FET], 4-borono-2-^18^F-fluorophenylalanine [FBPA]), FBY displayed lower background activity with maximum and mean SUV values of 0.116 and 0.037, respectively, allowing a clearer visualization of tumors and potentially aiding in the identification of small lesions as well as the delineation of treatment targets. This phenomenon may result from the replacement of the carboxyl group (-COOH) with the trifluoroborate group (-BF_3_) so that the molecule is not recognized as an amino acid and is not retained in the amino acid pool of tissues. The lower physiological uptake and higher T/N ratio of FBY compared with 4-boronophenylalanine (BPA) and sodium borocaptate (BSH) also protects the normal brain from redundant exposure during BNCT. In addition, FBY displayed higher stability (98% intact over 4 h of H_2_O_2_ incubation) than BPA (99% conversion at 1 h of H_2_O_2_ incubation) [6], and the same chemical structure can be simultaneously applied for diagnosis and therapy, highlighting its advantage over the current BNCT agents.

Currently, the extent of tumor remains delineated on MRI, with GTR defined by the T1-weighted contrast-enhanced regions for significant contrast-enhanced tumors [10]. However, biopsy-based studies indicated that tumor infiltrates beyond contrast enhancement [3, 13, 14], and a large-scale retrospective report suggested that the maximum resection of both contrast-enhanced and noncontrast-enhanced regions contributes to survival benefits in certain populations [15]. The identification of tumor infiltration beyond GTR is crucial for surgery, radiation planning and drug treatment [15, 16]. FBY, therefore, may aid in the accurate delimitation of the active tumor area since LAT-1 not only is expressed in the core tumor but also exists in the infiltrating tumor [17, 18]. In our study, the contrast-enhanced regions mostly displayed elevated FBY activity (mean overlap volume of 0.995), and the minimum contrast enhancement without FBY uptake may be the result of discordance of spatial resolution, where thin-slice MR images have higher resolution than FBY PET images in the x, y and z axes. Moreover, in accordance with the biological feature that diffuse glioma grows infiltratively while metastatic tumor, meningioma and pleomorphic xanthoastrocytoma has a clear tumor boundary, diffuse gliomas exhibited a larger active volume beyond contrast enhancement than circumscribed tumors, indicating that the FBY uptake outside of contrast enhancement was not the result of different resolution or preprocessing but may be suggestive of a true tumor boundary. The SUVmean for the regions that have increased FBY activity but not contrast enhancement were the same (while diffuse tumors have larger volumes), also supporting a true extension of FBY uptake rather than the result of resolution. Hence, FBY PET provides an alternative approach to delineate the active tumor that extends beyond MRI contrast enhancement and facilitates precision surgical and radiation planning to improve patient outcomes. Nevertheless, further multitarget biopsy-based investigations are essential to reveal the performance of FBY in the identification of infiltrating tumors [19].

The uptake pattern of FBY would also promote BNCT, since both contrast-enhanced and noncontrast-enhanced tumors display increased FBY uptake and can be included as treatment. In addition to the high T/N ratio in the core tumor, the infiltrative noncontrast-enhanced tumor also displayed sufficient FBY activity (SUVmean to Nmax of 5.87 ± 1.67, higher than the general requirement of a T/N ratio greater than 3.0–5.0), showing potential for treating infiltrative tumors to prevent regional recurrence. Nevertheless, it should be noted that LAT-1, a transporter for FBY and BPA, is saturable and may hinder the accumulation of these molecules when a larger chemical quantity is needed for BNCT [20, 21]. Although FBY in preclinical studies and BPA in clinical trials demonstrated the efficacy of LAT-1-based boron carriers [6, 22], whether FBY delivers sufficient boron under therapeutic doses in clinical scenarios requires further investigation.

In conclusion, FBY uptake beyond contrast enhancement is more significant in diffuse tumors than in circumscribed tumors, which may aid the delineation of active tumor areas and facilitate BNCT.

## Data Availability

The data included in the study are available from the corresponding author upon reasonable request.
